# Author Correction: RNA editing of BFP, a point mutant of GFP, using artificial APOBEC1 deaminase to restore the genetic code

**DOI:** 10.1038/s41598-024-71985-0

**Published:** 2024-09-09

**Authors:** Sonali Bhakta, Matomo Sakari, Toshifumi Tsukahara

**Affiliations:** Area of Bioscience and Biotechnology, School of Materials Science, Japan Advanced Institute of Science and Technology, M1‑4F, 1‑1 Asahidai, Nomi City, Ishikawa 923‑1292 Japan

Correction to: *Scientific Reports* 10.1038/s41598-020-74374-5, published online 14 October 2020

The original version of this Article contained an error in the sequence of the macular mutation.

As a result, Figure 3 and its legend have been updated.

The incorrect version of Figure 3 and its legend appear below:

**Figure 3:**
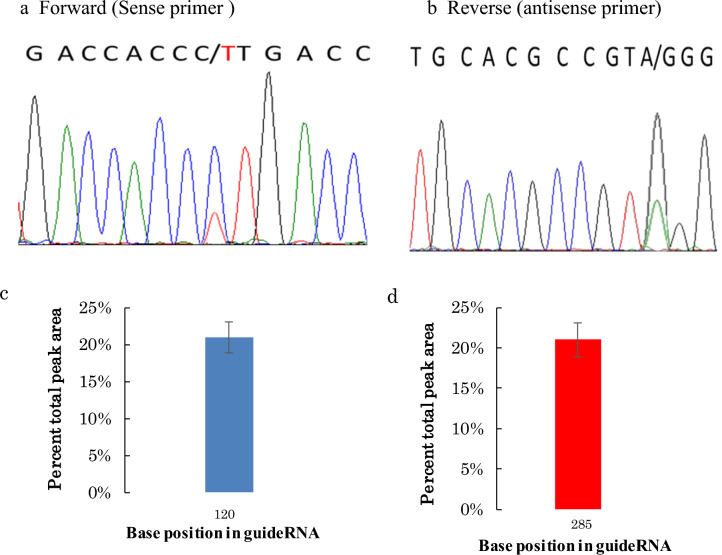
Confirmation of the restoration of BFP to GFP (C to U) by Sanger’s sequencing. (**a**) Forward or Sense primer (CCA to CTA). (**b**) Reverse or Antisense primer (GGG to AGG). In both the sense and antisense primers the dual peaks were observed, which were due to the restoration of the genetic code from the C to U (BFP to GFP), after the application of the two editing factors (APOBEC1 deaminase and gRNA). (**c**) and (**d**) Edit-R analysis of the Sense and antisense chromatogram height of the edited part of the peak and the statistical analysis (mean ± SEM) has been done, where the n = 5. Edit R Version 10 (https://moriaritylab.shinyapps.io/editr_v10/) analysis has been done by using the Sanger’s sequencing Ab.1 file.

The legend of Figure 3 now reads:

“Confirmation of the restoration of BFP to GFP (C to U) by Sanger’s sequencing. (**a**) Forward or Sense primer (CCA to CTA), the dual peaks were observed, which were due to the restoration of the genetic code from the C to U (BFP to GFP), after the application of the two editing factors (APOBEC1 deaminase and gRNA). (**b**) Edit-R analysis of the Sense and antisense chromatogram height of the edited part of the peak and the statistical analysis (mean ± SEM) has been done, where the n = 5. Edit R Version 10 (https://moriaritylab.shinyapps.io/editr_v10/) analysis has been done by using the Sanger’s sequencing Ab.1 file.”

In addition, Supplementary File 1 contains a new descriptive Figure S6.

The incorrect version of Supplementary File 1 appears below.

The original Article has been corrected.

## Supplementary Information

Supplementary Information.

