# Supplementary material for: Author Correction: RNA editing of BFP, a point mutant of GFP, using artificial APOBEC1 deaminase to restore the genetic code
Source: Sci Rep. 2024 Sep 9;14:20969. doi: 10.1038/s41598-024-71985-0 (PMC11385551; doi:10.1038/s41598-024-71985-0)

RNA editing of BFP, a point mutant of GFP, using artificial APOBEC1 deaminase to restore the genetic code

Sonali Bhakta1, Matomo Sakari1, and Toshifumi Tsukahara*1

Supplementary Data 1, S1:

List of candidate genes involved in diseases caused by T-to-C mutations

RNA editing of BFP, a point mutant of GFP, using artificial APOBEC1 deaminase to restore the genetic code

Sonali Bhakta1, Matomo Sakari1, and Toshifumi Tsukahara*1

Supplementary Data 2, S2:

RNA-sequencing (RNA-seq) reads from BFP_1 HEK 293

RNA editing of BFP, a point mutant of GFP, using artificial APOBEC1 deaminase to restore the genetic code

Sonali Bhakta1, Matomo Sakari1, and Toshifumi Tsukahara*1

Supplementary Data 3, S3:

RNA-sequencing (RNA-seq) from experimental HEK 293_1

RNA editing of BFP, a point mutant of GFP, using artificial APOBEC1 deaminase to restore the genetic code

Sonali Bhakta[1], Matomo Sakari[1], and Toshifumi Tsukahara*[1]

Supplpementary Data 4, S4:

Calculation of editing efficiency based on peak area and peak height of the Sanger sequencing (N=5)

	**Height**	**mean height**	**Width**	**Mean Width**	**Mean area**	**Mean total**	**STDv of Height**	**STDv of width**		**Ratio of edited and unedited**			**From the peak height**
Original C	431		48										
	384		59										
	410	406.2	38	47.6	19335.12		17.824	8.142					
	395		42										
	411		51										0.21
						23490				0.215	21.5		21%
Edited T	120		42								21.5%		
	110		35										
	95	108.2	38	38.4	4154.88		9.859	3.049					
	102		36										
	114		41										

RNA editing of BFP, a point mutant of GFP, using artificial APOBEC1 deaminase to restore the genetic code

Sonali Bhakta[1], Matomo Sakari[1], and Toshifumi Tsukahara*[1]

Supplementary Figure 5, S5:

Comparison of editing efficiency and off target effects when using gRNA of different lengths and different positions of the target in the guide

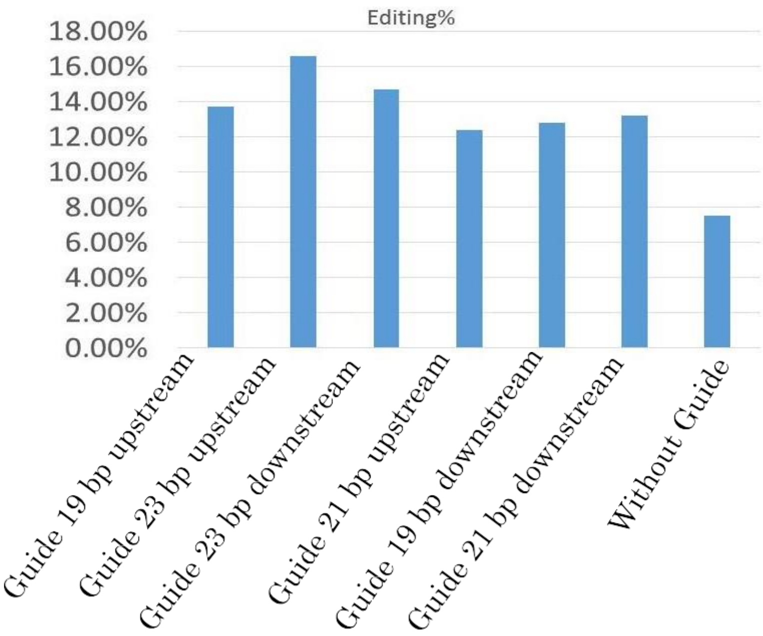

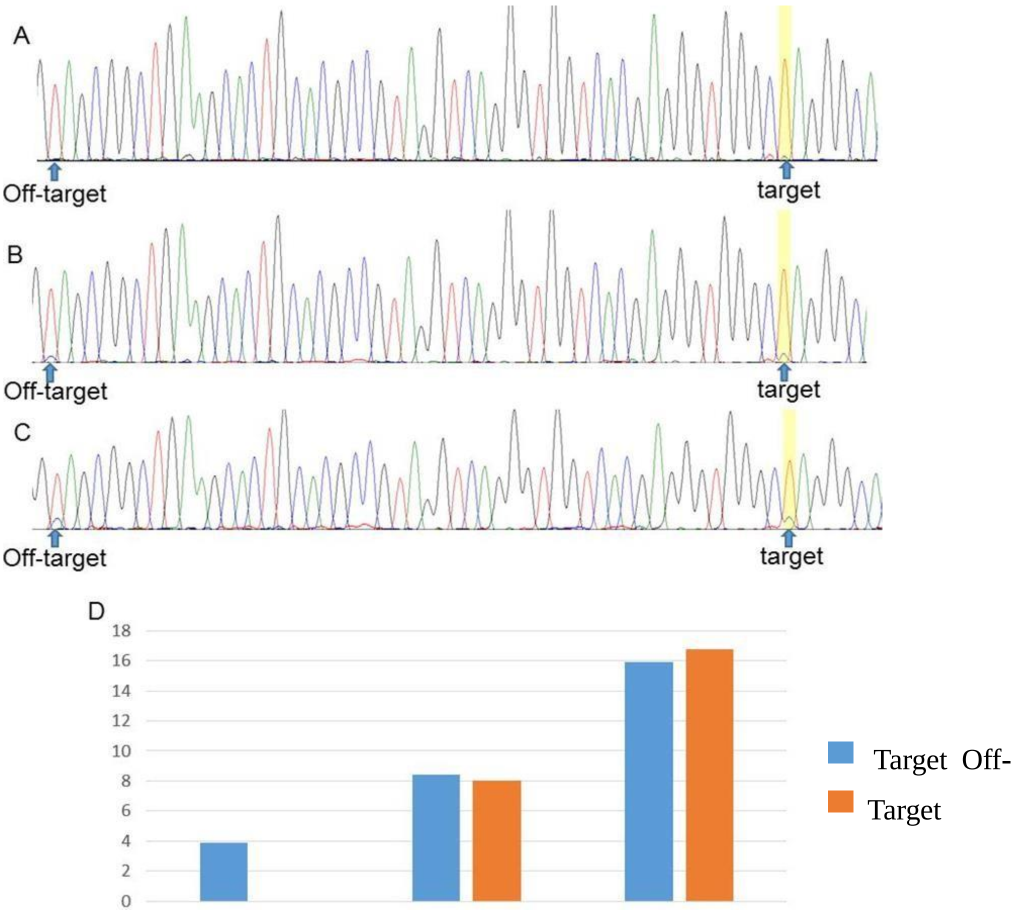

ADAR1-DD 250 ng Guide 19 bp upstream 500 ng

ADAR1-DD 250 ng Guide 21 bp upstream 500 ng

ADAR1-DD 250 ng Guide 23 bp upstream 500 ng

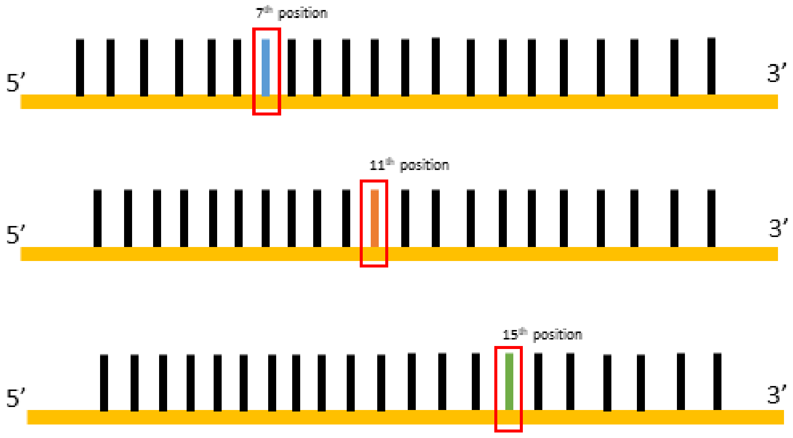
7th position
11th position
15th position
5'
3'

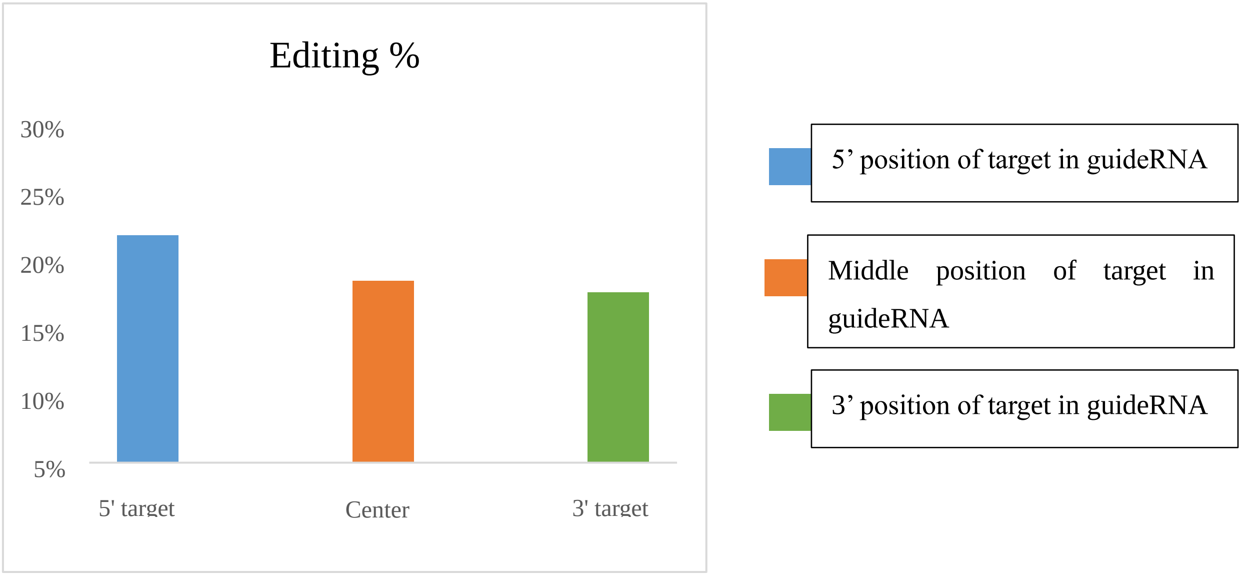
Editing %
30%
25%
20%
15%
10%
5%
5' target
Center
3' target
5' position of target in guideRNA
Middle position of target in guideRNA
3' position of target in guideRNA

RNA editing of BFP, a point mutant of GFP, using artificial APOBEC1 deaminase to restore the genetic code

Sonali Bhakta[1], Matomo Sakari[1], and Toshifumi Tsukahara*[1]

Supplementary Data 6, S6:

Mutation reads at different positions in BFP stably transformed in HEK 293 cells- target/control

Pattern	Count
T>G	1536
A>C	1469
A>G	1107
T>C	921
G>T	634
C>A	625
T>A	534
A>T	481
G>A	405
C>T	396
G>C	361
C>G	298

Pattern	Count
AA>GG	69
TT>CC	41
GA>TT	17
AG>CC	15
CT>GG	12
AG>TT	9
GA>CC	9
CC>GG	6
TC>AA	5
AA>CC	5
CT>AA	5
TC>GG	5
AT>TG	4
GG>CC	4
GT>CG	4
AC>CG	4
AAA>GGG	3
TG>AT	3
TT>AA	3
GG>AA	3
AT>CG	3
AT>CC	3
TT>CG	3
CG>AC	2
GC>TA	2
CA>AT	2
AT>CA	2
TA>GG	2
CA>TG	2
GA>TC	2
GG>TT	2
CC>TG	2

Pattern	Count
TTT>AAA	1
TA>CC	1
TT>GG	1
TTT>CCC	1
TC>GA	1
GAA>CGG	1
AA>TT	1
TC>CT	1
AT>GG	1
TC>GT	1
CT>GC	1
AAAA>GGCC	1
CTC>AAA	1
CT>TA	1
AT>GA	1
ATC>CAA	1
CAC>TGG	1
TG>GC	1
TC>CG	1
AC>TG	1
CG>GC	1
AGA>TTT	1
AC>CA	1
GA>AG	1
CC>TT	1
GAT>TTG	1
GA>AC	1
TG>CC	1
GA>AT	1
GT>AG	1
CA>GG	1
GT>CC	1
AA>CG	1

.

RNA editing of BFP, a point mutant of GFP, using artificial APOBEC1 deaminase to restore the genetic code

Sonali Bhakta[1], Matomo Sakari[1], and Toshifumi Tsukahara*[1]

Supplementary Data 7, S7:

Mutation reads at different positions in restored GFP in HEK 293 cells-tested/ experimental

**Pattern**	**Count**
T >G	2401
A >C	2355
T >C	1972
A >G	1915
G >T	1745
C >A	1698
G >A	1271
C >T	1238
T >A	1134
A >T	1042
C >G	702
G >C	648

**Pattern**	**Count**
GG>AT	1
TG>CC	1
TG>GT	1
CTG>AAT	1
TA>AT	1
CG>GC	1
AAAA>TTTT	1
CC>AA	1
CA>GG	1
GC>CA	1
CA>GT	1
TC>AT	1
CCT>GGG	1

**Pattern**	**Count**
AA >GG	58
TT >CC	51
GA >TT	31
TC>AA	23
CT >GG	20
AG >CC	16
AA >CC	14
AG >TT	12
AT >CA	10
AT >TG	10
CT>AA	9
TC>GG	8
CA>TG	8
TG>CA	8
GG>CC	7
CC>GG	6
AC>CG	6
GA>CC	5
TT>CG	5
GT>>CG	4
CA>AT	4
CA>GC	3
AC>GT	3
CT>TC	3
TA>GG	3
AAA>GG G	3
AG>GC	3
AA>TT	2
AT>CC	2
TC>CT	2
TA>AC	2
AT>GG	2
GG>TT	2

**Pattern**	**Count**
AAA>TTT	2
TTT>CCC	2
CT>GC	2
GT>AA	2
GC>AA	2
AG>AA	2
CA>TC	2
GC>CT	2
GC>AT	2
GC>TT	2
GG>AA	2
TA>CC	2
AA>TG	2
GT>AC	2
GTT>CCG	2
TG>AC	2
AT>CG	2
AG>CA	2
CC>TA	22
TG>AT	2
TG>AA	2
TG>CT	2
CT>TA	1
CC>TG	1
ACA>TTT	1
GCT>TGG	1
CG>AA	1
CT>AC	1
TT>GG	1
GA>TG	1
GA>AG	1
CTCT>GCGG	1
CT>GA	1

**Pattern**	**Count**
AA>CT	1
CTC>AAA	1
TT>AA	1
GAT>TTG	1
GC>TG	1
CG>AT	1
GA>TC	1
GTT>CGG	1
CC>AG	1
TT>AG	1
TC>GA	1
AAG>GTT	1
GT>CC	1
TCG>AAA	1
TA>CG	1
GGG>CCC	1
AG>GT	1
CC>GT	1
AGT>CTC	1
CT>AG	1
AGA>CCC	1
CTC>GGG	1
AGT>CCG	1
TCT>AAA	1
TA>GT	1
CA>AC	1
AC>TG	1
CCCA>GGGT	1
TT>CA	
GT>TC	1
ATC>CAA	1
TT>GA	1
GT>CA	1

RNA editing of BFP, a point mutant of GFP, using artificial APOBEC1 deaminase to restore the genetic code

Sonali Bhakta[1], Matomo Sakari[1], and Toshifumi Tsukahara*[1]

Supplementary Figure 8, S8

Schematic diagram of the editing by guideRNA (Complementary to the target sequence) and APOBEC 1 deaminase

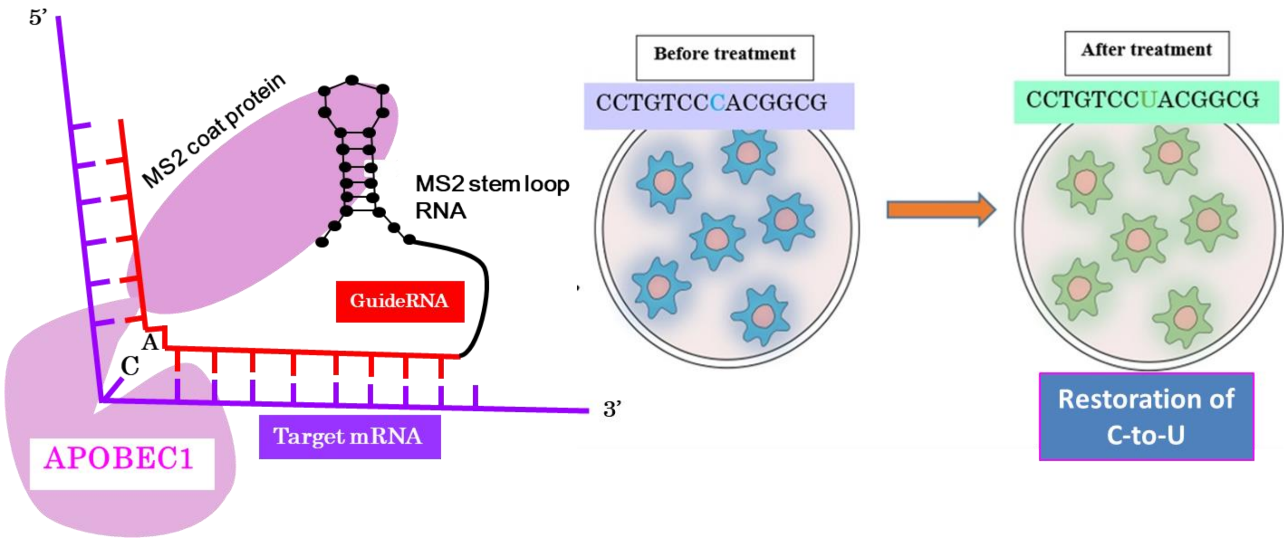

RNA editing of BFP, a point mutant of GFP, using artificial APOBEC1 deaminase to restore the genetic code

Sonali Bhakta1, Matomo Sakari1, and Toshifumi Tsukahara*1

Supplementary Data 9, S9:

PCR-RFLP raw gel image without cropping

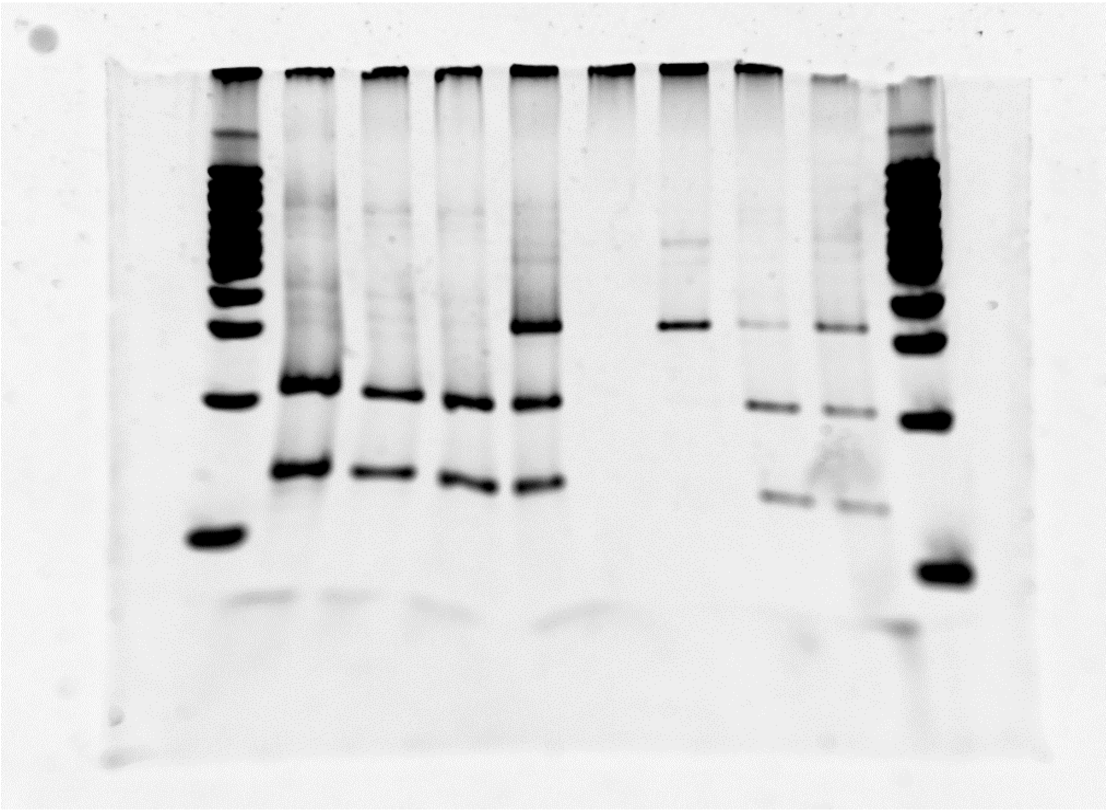

Supplement: Supplementary file 1 — Supplementary Information. [file 41598_2024_71985_MOESM1_ESM.pdf]
